# Complete chloroplast genome of *Prunus rufa* (Rosaceae), a wild flowering cherry endemic to the Himalayan region

**DOI:** 10.1080/23802359.2019.1698363

**Published:** 2019-12-13

**Authors:** Yue-Ling Li, Barry Clarke, Jian-Hui Li, Zhong-Shuai Sun

**Affiliations:** a Zhejiang Provincial Key Laboratory of Plant Evolutionary Ecology and Conservation, Taizhou University, Taizhou, Zhejiang, China;; b The Sir Harold Hillier Gardens, Romsey, United Kingdom;; c College of Chemistry and Materials Engineering, Quzhou University, Quzhou, Zhejiang, China

**Keywords:** *Prunus rufa*;·*Prunus sensu lato*, chloroplast genome, phylogenomics

## Abstract

*Prunus rufa* is a species of Prunus native to the Himalayan region. We determined the first complete chloroplast genome of *P. rufa* using a genome skimming approach. The cp genome was 157,723 bp long, with a large single-copy region (LSC) of 85,860 bp and a small single-copy region (SSC) of 19,081 bp separated by a pair of inverted repeats (IRs) of 26,391 bp each. It encodes 129 genes, including 84 protein-coding genes, 37 tRNA genes, and 8 ribosomal RNA genes. We also reconstructed the phylogeny of *Prunus sensu lato* using maximum-likelihood (ML) method, including our data and previously reported cp genomes of related taxa. The phylogenetic analysis indicated that *P. rufa* is closely related to *Prunus cerasoides*.

*Prunus rufa* Hook.f. is a wild cherry species of Prunus endemic to the Himalayan region, extending from Nepal, E Himalaya to Tibetan Plateau (Ohba and Akiyama 2010; Ohba et al. 2012). The classification of the *Prunus sensu lato* (Rosaceae) has long been problematic; phylogenetic studies using a limited set of markers have often not been able to fully resolve relationships within this genus, indicating that a higher number of molecular characters are required for an improved understanding of relationships within this group (Shi et al. 2013; Chin et al. 2014). By taking advantage of next-generation sequencing technologies that efficiently provide the chloroplast (cp) genomic resources of the species under study, we can rapidly access the abundant genetic information for phylogenetic research and conservation genetics (Liu et al. 2017, 2018). Therefore, we sequenced the whole chloroplast genome of *P. rufa* to elucidate its phylogenetic relationship with other *P. sensu lato*.

Total genomic DNA was extracted from silica-dried leaves collected from the living collections (accession no: 1987.0221) of the Sir Harold Hillier Gardens (Romsey, Hampshire, United Kingdom) using a modified CTAB method (Doyle and Doyle 1987). A voucher specimen (Clarke1805001) was collected and deposited in the Herbarium of Taizhou University. DNA libraries preparation and pair-end 125 bp read length sequencing were performed on the Illumina HiSeq 2500 platform. About 10.5 GB of clean data were trimmed and assembled into contigs using CLC Genomics Workbench 8. All the contigs were then mapped to the reference cp genome of *Prunus speciosa* (Koidz.) Nakai (MH998233; Sun et al. 2019) using BLAST (NCBI BLAST v2.2.31) search and the draft *cp* genome of *P. rufa* was constructed by connecting overlapping terminal sequences in Geneious R11 software (Biomatters Ltd., Auckland, New Zealand). Gene annotation was performed via the online program Dual Organellar Genome Annotator (DOGMA; Wyman et al. 2004).

The complete cp genome of *P. rufa* (GenBank accession MN648456) was 157,723 bp long consisting of a pair of inverted repeat regions (IRs with 26,391 bp) divided by two single-copy (SC) regions (Large SC[LSC] with 85,860 bp; small SC [SSC] with 19,081 bp). The overall GC contents of the total length, LSC, SSC, and IR regions were 36.7, 34.6, 30.2, and 42.5%, respectively. The genome contained a total of 129 genes, including 84 protein-coding genes, 37 tRNA genes, and 8 rRNA genes.

We used a total of 20 additional complete cp genomes of the *P. sensu lato* species to clarify the phylogenetic position of *P. rufa*. *Prunus serotina* Ehrh. (NC036133) and *Prunus padus* L. (NC026982) in Subg. *Padus* was used as the outgroup. We reconstructed a phylogeny employing the GTR + G model and 1000 bootstrap replicates under the maximum-likelihood (ML) inference in RAxML-HPC v.8.2.10 on the CIPRES cluster (Miller et al. 2010). The ML tree (Figure 1) was consistent with the most recent phylogenetic study on *P. sensu lato* (Shi et al. 2013; Chin et al. 2014). *Prunus rufa* exhibited the closest relationship with *Prunus cerasoides* D. Don.

**Figure 1.**
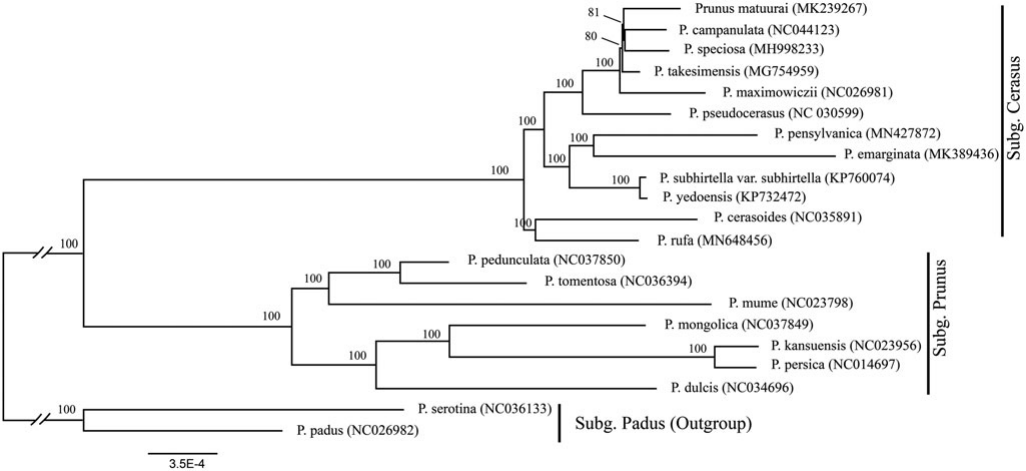
Phylogenetic tree reconstruction of 21 taxa of *Prunus sensu lato* using maximum-likelihood (ML) method. Relative branch lengths are indicated. Numbers near the nodes represent ML bootstrap value. The scientific names of some species are debated.
